# Advances in the diagnosis and management of post-stroke limb spasticity: a narrative review

**DOI:** 10.3389/fneur.2026.1781709

**Published:** 2026-04-13

**Authors:** Menghan Su, Mingqing Yu, Ping Xu, Yong Fei

**Affiliations:** 1 Zhejiang Chinese Medical University, Hangzhou, Zhejiang, China; 2 Department of Anesthesiology and Pain, The Affiliated Hospital of Jiaxing University, Jiaxing, Zhejiang, China

**Keywords:** botulinum toxin, limb spasticity, neuromodulation, post-stroke sequelae, rehabilitation

## Abstract

**Objective:**

This review aims to provide a comprehensive overview of the current understanding and clinical management of post-stroke limb spasticity.

**Methods:**

Literature searches were conducted in PubMed and CNKI databases for articles published between January 2000 and June 2025, using keywords related to post-stroke spasticity, assessment, and interventions (botulinum toxin, rehabilitation, neuromodulation, surgery). The search was restricted to English-language articles addressing post-stroke limb spasticity. After screening, 68 studies were included and categorized by research theme.

**Results:**

Several interventions were identified as effective in alleviating post-stroke limb spasticity. Specifically, botulinum toxin injection has emerged as the primary choice for managing focal spasticity. Neuromodulation methods, including transcranial magnetic stimulation, transcranial direct current stimulation, spinal cord stimulation, and vagus nerve stimulation, demonstrated significant therapeutic potential.

**Conclusion:**

Neuromodulation techniques exert their effects by adjusting corticospinal tract excitability and promoting neural plasticity. However, additional randomized controlled trials are necessary to optimize stimulation settings and confirm their long-term effectiveness.

## 1 Introduction

Stroke is one of the leading causes of disability and death worldwide (1). Survivors often face a range of long-term and disabling sequelae, including motor dysfunction, speech and swallowing disorders, cognitive impairment, psychiatric sequelae, and post-stroke epilepsy (2). Post-stroke spasticity (PSS), as a common manifestation of motor disorders following upper motor neuron injury, is a critical pathological factor that hinders functional recovery in patients, increases the difficulty of care, and leads to pain and secondary complications such as joint contractures and pressure sores. In 1980, Lance first proposed the definition of spasticity. He defined spasticity as a velocity-dependent increase in muscle tone resulting from damage to the upper motor neurons (3). This theory holds that spasticity is caused by abnormal reflex regulation at the spinal cord level after injury to upper motor neurons. With the progress of research evidence, Pandyan et al. (2005) (4) proposed a new definition of spasticity. They described spasticity as a disorder of sensorimotor control, manifested as intermittent or continuous involuntary activation of muscles. It emphasizes that the essence of spasticity is an imbalance in the regulation of sensory input and motor output, rather than merely an abnormal reflex. This explains why muscle tension still occurs at rest and also indicates the contribution of spasticity to movement disorders. Li et al. (2021) (5) further clarified the pathophysiological mechanism of post-stroke spasticity by combining clinical studies on stroke patients with animal experimental evidence. When the motor cortex or corticobulbar tract is injured, the inhibitory effect on the brainstem descending pathways disappears, resulting in excessive excitation of spinal motor neurons and stretch reflex circuits. This “de-inhibition” state eventually leads to an increase in muscle tone. Spasticity coexists with abnormal synergistic movement, muscle co-activation, and other movement disorders, all of which originate from abnormal changes in neural plasticity after upper motor neuron injury. Post-stroke spasticity typically occurs within weeks to months following a stroke (6), primarily affecting the muscle groups of the upper and lower limbs. This condition is characterized by increased muscle tone in the affected muscle groups, ultimately leading to limb stiffness, restricted movement, and involuntary muscle contractions. This pathological condition can significantly limit the patient’s ability to move independently, leading to limb deformities and pain. It places a heavy burden on the patient’s daily activities, self-care capabilities, and caregivers, severely impacting the quality of life of the patients (7–10). If left untreated, prolonged spasms can progress to severe joint contractures and impaired motor coordination, potentially leading to permanent disability. Therefore, early identification, intervention, and management of post-stroke limb spasticity are crucial. However, statistics show that more than one-third of stroke survivors still experience spasticity 12 months after the stroke (6). Among nursing home populations, the prevalence of upper limb spasticity in stroke survivors reaches as high as 70% (11), which indicates a significant unmet need for clinical management of spasticity (12, 13).

The complexity and multifactorial nature of spasticity present numerous challenges in its diagnosis and treatment. Currently, commonly used clinical assessment methods, such as the Modified Ashworth Scale (MAS) and the Tardieu Scale, while convenient and straightforward, have limitations including strong subjectivity, poor reliability, and ceiling effects. These limitations make it challenging to objectively quantify the severity of spasticity and conduct an in-depth analysis of the underlying pathophysiological mechanisms that lead to spasticity (14, 15). In terms of treatment, due to the complex mechanisms underlying post-stroke limb spasticity, numerous therapeutic approaches and studies currently exist, yet their efficacy varies significantly. There is a lack of unified standards and protocols. This review summarizes the literature on the diagnosis and treatment of post-stroke limb spasticity from January 2000 to June 2025 in the National Library of Medicine (PubMed) and the China National Knowledge Infrastructure (CNKI), with a focus on summarizing the applications of botulinum toxin, rehabilitation therapy, surgical interventions, and neuromodulation techniques in the management of spasticity.

## 2 Methodology

**Figure 1:**
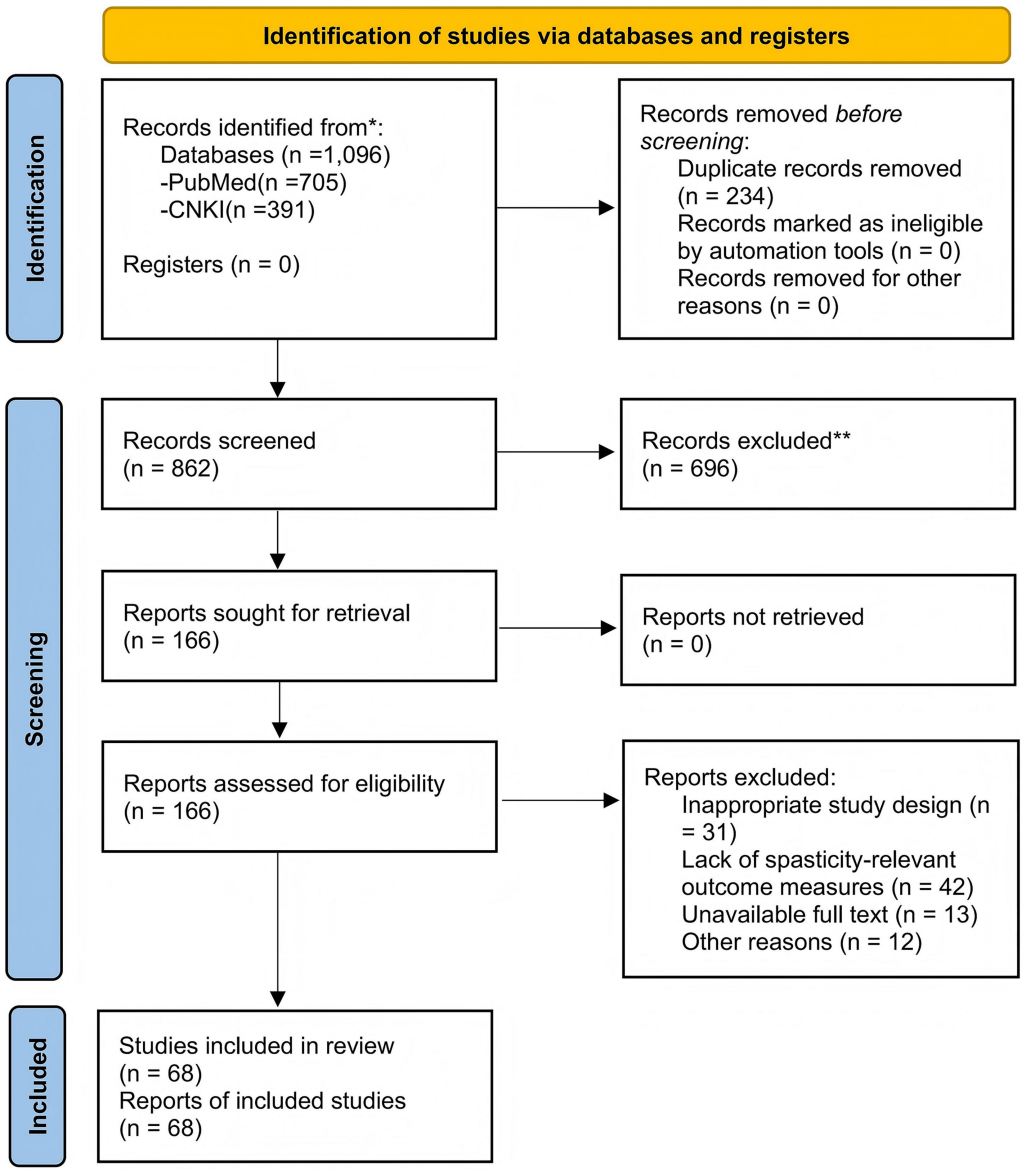
Flow chart of literature screening. The PRISMA flow diagram was adapted from (107).

We retrieved the literature from January 2000 to December 2025 in the PubMed and CNKI databases. The keywords used in the search included ‘stroke’, ‘spasticity’, ‘botulinum toxin A’, ‘physical therapy’, ‘neuromodulation’, ‘surgical intervention’ and ‘rehabilitation’. Inclusion criteria: (1) Research on intervention measures for spasm or motor recovery after stroke, original research or systematic review/meta-analysis, (2) published in peer-reviewed journals, (3) publication time between 2000 and 2025, in English or Chinese. Exclusion criteria: (1) Repeatedly published research; (2) It is not related to the topic of the review; (3) Low-quality studies with obvious methodological flaws (unless limitations are discussed). (4) Conference abstracts, letters, editorials, animal experiments/preclinical studies without original data (unless mechanisms of action are discussed). Two authors independently screened the titles/abstracts. When there was a disagreement, a third author joined in the evaluation and discussion. Use EndNote X9 and manual validation to delete duplicate references. Preliminary search: 1096 records (705 on PubMed, 391 on CNKI). After eliminating 234 duplicates, 826 were screened out and 660 were excluded. A full-text assessment of 166 articles led to the exclusion of 98. Ultimately, 68 studies were included (42 original studies and 26 systematic reviews/meta-analyses). Although the included RCT studies were not rated for quality, the quality ratings of the cited systematic reviews/meta-analyses were reported in detail in this paper. The selection process is shown in Figure 1.

### 2.1 Pathophysiological mechanisms and objective assessment

Post-stroke limb spasticity results from the complex remodeling of neural pathways and functional abnormalities that occur following upper motor neuron injury. The core mechanism involves increased spinal cord excitability, manifested as hyperreflexia (16, 17). This hyperactivity may be associated with increased excitability of spinal motor neurons, and potential sources include enhanced effects of other downstream pathways, such as the reticulospinal tract (RS) and vestibulospinal tract (VS), following diminished downstream inhibition of corticospinal tracts (17). Interestingly, the research found that the stretch reflex threshold in the affected limb of stroke survivors is decreased, suggesting an increase in the excitability of motor neurons. Conversely, the stretch reflex threshold in the unaffected limb is also higher than that of healthy controls. This supports the hypothesis that the bilateral projection pathways of the reticulospinal (RS) tract may partially contribute to the increased excitability of bilateral motor neurons following a stroke, even though clinically, spasticity primarily manifests in the affected side (17).

In addition to neural changes, spasticity also involves alterations in the biomechanical properties of muscles and connective tissues, such as abnormalities in muscle viscoelasticity (18, 19). These non-neural factors also contribute significantly to muscle stiffness and restricted movement (14). Additionally, abnormal muscle synergy patterns, such as flexor synergy, are common symptoms of motor dysfunction following a stroke and interact with spasticity (20, 21). It has been shown that the exaggerated motor overflow phenomenon is prevalent in stroke survivors with spasticity and may share a common pathophysiological process with spasticity, the mechanism of which may originate from subcortical structures (22). In terms of gait, patients with post-stroke hemiparesis often exhibit abnormal patterns of synergistic muscle contraction, particularly around the knee and ankle joints, which may serve as a compensatory strategy to enhance gait stability but may also be associated with spasticity and lead to increased metabolic costs (23).

Currently, the most prevalent methods for evaluating spasms in clinical practice encompass scale assessments, electrophysiological measurements, and biomechanical evaluations. These traditional assessment techniques primarily focus on qualitative or semi-quantitative observations. Commonly utilized scales for spasm evaluation include the modified Ashworth scale, which measures changes in resistance during passive movement, as well as the modified Tardieu Scale (MTS) and the Composite Spasm Scale (CSS) (24), both of which assess spasms based on varying stretching speeds. The results obtained from the Modified Ashworth Scale are subject to the assessor’s subjectivity, the patient’s level of cooperation, and the inherent characteristics of the muscle, leading to criticisms regarding its reliability. In contrast, the modified Tardieu Scale offers a more precise reflection of spasm characteristics, specifically the increase in muscle tone that is speed-dependent. Nonetheless, this assessment method is contingent upon the assessor’s experience and is less frequently employed in clinical settings. Additionally, some studies have indicated that the modified Tardieu Scale suffers from inadequate repeatability, with only 25 studies validating its repeatability (25). Neuroelectrophysiological methods and biomechanical measurements provide more objective and accurate assessment approaches. Electrophysiological techniques, including surface electromyography (sEMG) and action evoked potential (MEP), offer objective indicators for understanding the neurophysiological mechanisms underlying spasms by measuring the electrical activity and nerve conduction in muscles. In the assessment of spasms, sEMG primarily identifies characteristics of abnormal muscle activity, such as reflexive muscle contractions, variations in the amplitude of electromyographic signals, and irregular frequency components (26, 27). Motor evoked potentials (MEPs) are elicited by single-pulse transcranial magnetic stimulation (TMS) applied to the motor cortex, which activates corticospinal neurons and produces a recordable electromyographic response in target muscles. Motor evoked potential (MEPs) parameters, including motor threshold (MT), amplitude, latency, and central motor conduction time (CMCT), are established neurophysiological biomarkers for evaluating the integrity of the corticospinal tract and predicting motor recovery potential after stroke (28). The presence of MEPs indicates preserved corticospinal connectivity and is associated with better functional outcomes, while absent or abnormal MEPs parameters correlate with more severe motor impairment. The reliability of MEPs measurement depends on the key technical parameters of transcranial magnetic stimulation (TMS), including the placement accuracy of the coil relative to the target cortex, the orientation Angle, and the stimulation intensity based on the resting or active motion threshold. These factors must be carefully controlled to ensure reproducible assessment outcomes. Biomechanical techniques, including Isokinetic Dynamometry and the Pendulum Test, can quantify muscle stiffness and abnormal movement patterns by measuring mechanical parameters during limb motion. Isokinetic muscle strength testing employs specialized isokinetic testing instruments. A feedback system adjusts the resistance to maintain a constant angular velocity of muscle contraction throughout joint movement. Concurrently, parameters such as torque, power, and work performed by the muscle are recorded to facilitate a quantitative assessment of muscle function. The pendulum test entails releasing the patient’s legs from a horizontal position and recording kinematic parameters during their free swinging, including swing amplitude, speed, number of swings, and Relaxation Index (RI) (29). To maintain the objectivity of the measurement method, it is often essential to incorporate specialized testing equipment or sensor technology. The complexity of operation and the high cost of such equipment restrict their application in clinical practice.

Due to the inherent subjectivity and constraints of clinical evaluation methods, there is a growing emphasis on developing an objective and quantitative spasm assessment system. These new systems typically integrate various sensor technologies to collect kinematic, biomechanical, and electrophysiological information simultaneously. A recent study introduced an evaluation system utilizing exoskeleton measurement devices to quantify upper limb spasm severity (14, 18). This system is capable of recording kinematic, biomechanical, and surface electromyography (sEMG) signals during passive distraction movements. Mechanical impedance (reflecting viscoelastic changes) and electromyographic signal characteristics (reflecting neurophysiological mechanisms) of the joint can be recognized by means of improved genetic algorithms or multilayer processing models (18). Subsequent research has leveraged long short-term memory networks (LSTM) and parallel models to integrate pathological electrophysiological results with kinematic deviations and biomechanical attributes, thereby distinguishing pathological variations at both neural and non-neural levels and furnishing comprehensive quantified assessments of spasticity severity (14). These systems have exhibited robust reliability in discriminating spastic hypertonia from normal muscle tone, with the quantitative evaluation outcomes displaying a significant correlation with clinical rating scales (14, 18).

The development of these objective assessment systems provides clinicians with more reliable tools, which are helpful for the diagnosis of spasticity, disease monitoring, and adjustment of treatment plans (14, 18). Their ability to capture details that are difficult to quantify on clinical scales, such as distinguishing between neural and non-neural components of spasticity or recognizing abnormal patterns in active movement, is of significant importance for developing more targeted rehabilitation strategies. However, most of these systems are in the research phase, and their usability, cost, and broader applicability in clinical settings still require further validation.

### 2.2 New advances in the treatment of post-stroke limb spasticity

Post-stroke limb spasticity is characterized by its complexity and multifactorial nature, and the underlying pathophysiological mechanisms remain unclear. There are numerous treatment methods for spasticity; however, a unified consensus is lacking. In recent years, various therapeutic approaches, including pharmacological interventions, physical rehabilitation, surgical procedures, and neuromodulation, have made new advancements, emphasizing the advantages of a comprehensive application.

## 3 Drug treatment

Botulinum toxin type A (BoNT-A) is the first-line pharmacological treatment for focal post-stroke spasticity (30–32). It produces temporary, reversible chemodenervation by cleaving synaptosome-associated protein-25 (SNAP-25) and inhibiting acetylcholine release at neuromuscular junctions (33, 34). Multiple studies have demonstrated BoNT-A efficacy in reducing upper and lower limb spasticity as measured by the Modified Ashworth Scale, as well as alleviating pain and improving quality of life (16, 31, 35–38). For lower extremity spasticity, a systematic review and meta-analysis identified that for the most common pattern (spastic plantar flexors), the optimum dose is medium-dose (approximately 300 U Botox® or 1,000 U Dysport®) (39). Recent evidence suggests that higher doses may be safe in appropriately selected patients. A 2025 retrospective study found that increasing onabotulinumtoxinA dose from 400 U to 600 U resulted in no adverse events, with all patients reporting improved spasticity reduction (40). During the treatment, enhancing the precision of BoNT-A injections is crucial for optimizing efficacy and minimizing side effects. For this reason, various assisted injection techniques have been applied in clinical practice and experiments. Currently, the assisted injection techniques mainly include four types: anatomical landmarks and palpation localization, electrical stimulation (ES), electromyography (EMG), and ultrasound-guided visualization (US) (41, 42). Ultrasound-guided assisted injection technology is recommended as the optimal assistive technology because it enables precise localization of target muscles and their motor points, thereby improving treatment efficacy and potentially reducing dosage requirements (37, 41), although another study also have reported no difference in the therapeutic effect of ultrasound-guided and electrical stimulation-assisted injection on spasms (43). The timing of injection has garnered significant attention. Currently, no consensus exists regarding the optimal period for BoNT-A injection. Results from two longitudinal cohort studies indicate that early injection, specifically within three months post-stroke, may result in greater reductions in muscle tone in the short term (1–3 months). Furthermore, early intervention can lead to more rapid and substantial improvements in upper limb strength, sensorimotor recovery, and overall disability (44, 45). In clinical practice, it is advisable to consider botulinum toxin treatment immediately when spasms induce pain, compromise posture, hinder functional activities, or increase the difficulty of care (46).

Despite the significant effects of BoNT-A in reducing muscle tone, the extent to which it improves functionality remains a subject of debate (31, 47, 48). Some studies have found that the combination of BoNT-A and physical therapy can improve muscle tone, but it has not shown advantages over physical therapy alone in functional performance (such as walking speed and distance) (47, 48). However, other studies, particularly those focusing on patient-specific goal attainment (Goal Attainment Scale, GAS), have demonstrated that BoNT-A treatment can significantly enhance the rate of achieving functional goals for patients (38, 48). This suggests that improvements in functionality may be more evident in the individualized activities directly related to spasticity in patients, rather than in generic measures of motor function.

Botulinum toxin type A (BoNT-A) produces a temporary reduction in spasticity. After injection, the onset of clinical effect typically occurs within several days, the maximal clinical benefit is generally observed within the first few weeks, and the effect then gradually wanes over time. According to a systematic review and meta-analysis of lower extremity spasticity after stroke, significant improvement in muscle tone is usually achieved at the fourth and eighth weeks, and the therapeutic effect is maintained until the 12th week^39^. Therefore, repeat injections are commonly needed. Although 12 weeks is widely used as a reference interval for retreatment (30), the actual reinjection interval should be individualized and may be longer or shorter depending on clinical response, functional goals, formulation/dose, injected muscles, and patient factors. Long-term treatment with BoNT-A requires continuous monitoring and evaluation. A retrospective study found that although long-term therapy can consistently reduce muscle tone, the extent of improvement in certain walking parameters (such as the 6-min walking distance and the 10-meter walking speed) may diminish over time, while remaining stable in other parameters (such as the Timed Up and Go test) (49). This suggests that long-term management requires the optimization of collaborative rehabilitation strategies, adjusting treatment goals and expectations based on the patient’s baseline functional level. Although BoNT-A is the first-line medication recommended by current guidelines for focal spasticity, it still faces some problems in clinical practice. A nationwide retrospective study in France has shown that the utilization rate of BoNT-A among stroke survivors is relatively low, with many patients failing to receive repeated injections at the recommended frequency of every 3–4 months. This indicates a deficiency in the implementation of the treatment protocol (32). This treatment approach is constrained by factors such as high treatment costs, the need for repeated injections, and poor patient compliance.

## 4 Physical rehabilitation and combined therapy

In the management of spasms, physical rehabilitation is the cornerstone and is often used in combination with drugs such as BoNT-A (12, 30, 50). A multidisciplinary rehabilitation team comprising physical therapists, occupational therapists, physicians, and other professionals working collaboratively is the core force in delivering comprehensive spasticity management (12, 30, 50).

.Various physical therapy techniques have been employed to alleviate spasms and improve functionality:

### 4.1 Extracorporeal shock wave therapy (ESWT)

In recent years, it has been recognized as a safe and effective method for treating muscle spasms (51). Its potential mechanisms include the production of nitric oxide, reduction of motor neuron excitability, induction of neuromuscular transmission dysfunction, and direct effects on the rheological properties of muscles (51). Radial extracorporeal shock wave therapy (ESWT) is commonly used to treat muscle cramps, demonstrating efficacy when applied to the muscle belly or the tendon insertion site. The effects can last from 4 to 12 weeks (51). A randomized controlled trial found that the combination of BoNT-A injections with focused extracorporeal shock wave therapy (ESWT) for upper limb spasticity showed additional benefits in improving finger flexor spasticity, suggesting that ESWT may serve as an adjunct therapy to BoNT-A (52). However, the impact of ESWT on functional recovery remains inconclusive, with varying results compared to BoNT-A or in combination applications (51).

### 4.2 Robot-assisted training (RAT)

Robot-assisted therapy (RAT) enables high-intensity, repetitive, task-specific training that may enhance neuroplasticity and functional recovery when combined with BoNT-A for post-stroke spasticity (53). A recent systematic review by Facciorusso et al. (54) synthesized evidence from seven randomized controlled trials (*N* = 229) examining BoNT-A combined with RAT for post-stroke spasticity. Key findings included: (1) BoNT-A consistently reduced spasticity across all studies, but additional spasticity reduction with RAT versus conventional rehabilitation was inconsistent; (2) lower-limb trials demonstrated greater improvements in walking capacity and balance when RAT was added; (3) upper-limb trials showed comparable motor recovery across treatment arms, with occasional advantages in strength and movement quality; and (4) a pilot four-arm study suggested that initiating RAT approximately four weeks post-injection may maximize upper-limb motor gains. The review concluded that combining BoNT-A with RAT is safe and particularly promising for gait rehabilitation (54). A randomized controlled pilot trial by Hsieh et al. (55) compared BoNT-A combined with robot-assisted bimanual therapy (RBT) versus RBT integrating mirror therapy (RBMT) in 31 patients with chronic stroke and spastic fingers. Both groups showed significant improvements in FMA-UE, proximal motor function, MAS, and ARAT scores after 24 training sessions. Notably, only the RBMT group demonstrated significant improvements in distal FMA-UE and MAL scores, with gains maintained at 3-month follow-up. Between-group comparison favored RBMT for distal function at post-training, suggesting that combining robotic training with mirror therapy may enhance outcomes for finger spasticity (55). Another study comparing BoNT-A combined with robot-assisted therapy, mirror therapy, or active control training found that all three approaches improved motor function, reduced spasticity, and enhanced daily functioning; however, active control training may be more effective for long-term functional improvement (56). This suggests that the type and intensity of training may influence the outcome of the combined treatment.

### 4.3 Functional electrical stimulation (FES)

Functional electrical stimulation (FES) encompasses a range of techniques that apply electrical currents to elicit or assist functional movements (57, 58). A specific subtype, neuromuscular functional electrical stimulation (NMFES), targets antagonist muscles to inhibit spastic muscle activity via reciprocal Ia inhibitory pathways, thereby reducing spasticity and improving motor control (59). Several randomized controlled trials have demonstrated the effectiveness of FES in improving spasticity and balance in patients with hemiplegia (57, 58). For example, FES applied to ankle dorsiflexors during the swing phase of gait can reduce plantar flexor spasticity and improve walking (58). Huber et al. (59), evaluated NMFES combined with kinesiotherapy, specifically proprioceptive neuromuscular facilitation (PNF) techniques, on antagonist muscle activity in post-stroke patients. Kinesiotherapy refers to therapeutic exercise and movement-based rehabilitation aimed at improving motor control, strength, and functional performance. In this study, the control group received kinesiotherapy alone (PNF-based exercise therapy without electrical stimulation), while the experimental group received NMFES combined with the same kinesiotherapy protocol. The combined NMFES plus kinesiotherapy group demonstrated significantly greater reductions in spasticity, increases in muscle strength, and improvements in reciprocal antagonist muscle activation compared to the kinesiotherapy only group (59). These findings suggest that NMFES may modulate spinal motor centers, including the level of Ia inhibitory interneurons (59). However, subsequent meta-analyses have reported inconsistent findings regarding spasticity outcomes. Keesukphan et al. (60), conducted a network meta-analysis of 34 randomized controlled trials involving 1,476 stroke patients examining various stimulation therapies including FES, NMES, and their combinations (60). While FES significantly improved upper extremity function (pooled mean difference 3.61, 95% CI 0.14 to 7.07) and NMES plus TMS showed the highest probability for improving activities of daily living, none of the interventions demonstrated significant differences in spasticity reduction compared to conventional rehabilitation (60). The authors noted that the quality of evidence was low, highlighting the need for larger, rigorous trials (60).

### 4.4 Other physical therapies (dry needling and fascial manipulation)

Dry Needling has been proven to effectively alleviate post-stroke spasms and improve the accuracy of balance, range of motion, and stability maintenance. Incorporating it into the Bobath concept treatment plan can bring additional benefits (61). Fascial Manipulation (FM), as a novel approach, effectively alleviates lower limb spasms and ankle clonus in the short term by addressing abnormalities in connective tissues (fascia). It also improves the passive range of motion of the ankle joint and lower limb function, suggesting that fascia may play a role in the pathological processes of spasm (19).

Although various physical rehabilitation methods have shown potential, the current evidence remains unclear regarding which type, intensity, and setting of rehabilitation program is most effective following BoNT-A injections (50, 62). A feasibility randomized trial found that the addition of high-intensity rehabilitation after BoNT-A injections is feasible; however, it did not show significant advantages over either BoNT-A alone or rehabilitation alone in the short term. This suggests that larger-scale studies are needed to determine the actual benefits and optimal protocols for combined treatment (62).

## 5 Surgical intervention

Surgical intervention is an option for patients with spasticity who do not respond well to medications and conservative treatments, or who have severe limb deformities and joint contractures. Surgical methods include tendon lengthening or transfer, selective peripheral neurotomy (SPN), selective dorsal rhizotomy (SDR), and C7 nerve root transfer, among others.

### 5.1 Tendon lengthening or transfer

Tendon lengthening surgery alleviates spasticity and improves joint mobility by surgically cutting or lengthening tendons that have become excessively tight or shortened due to spasm, contracture, or scarring. In contrast, tendon transfer surgery restores muscle balance and function by repositioning or transferring the tendon to a new attachment point. A study investigated the functional improvement of 255 children with cerebral palsy who underwent lower limb tendon lengthening surgery after selective dorsal rhizotomy (SDR). With an average follow-up of 4.9 years post-surgery, 28 patients showed improvement in their Gross Motor Function Classification System (GMFCS) scores, while 213 patients maintained stable function. Notably, 81% of the children did not require further surgeries, indicating the significant long-term effects of this approach (63). However, the research team also pointed out that the age at the time of tendon lengthening surgery and the interval between SDR surgery and tendon lengthening surgery can have a significant impact on the risk of repeat surgery. When the interval between SDR surgery and tendon lengthening surgery is more than six months, the risk of repeat surgery will increase nearly threefold (63). In addition, some research reports have indicated that some patients have experienced complications such as nerve compression, overcorrection leading to deformity, and insufficient correction resulting in residual spasms after surgery. This has led to the need for further nerve forward movement or re-surgical correction (64, 65), which imposes a significant burden on the patients.

### 5.2 Selective peripheral nerve section (SPN)

Selective peripheral nerve section is applicable for focal spasms, as it reduces the excessive excitability of muscles and enhances spasm symptoms and functional status by severing the afferent fibers of certain peripheral nerves (66, 67). The SPN surgery alleviates symptoms by targeting specific nerves that innervate the spastic muscles. For the upper limbs, the surgery focuses on the nerves that innervate the pectoralis major and teres major, as well as the musculocutaneous nerve, median nerve, and ulnar nerve. For the lower limbs, it targets the obturator nerve, sciatic nerve, tibial nerve, common peroneal nerve, and femoral nerve (67). Multiple studies have demonstrated the effectiveness of SPN surgery in reducing spasticity (67–69). However, complications associated with the surgery, such as postoperative hematomas, transient sensory abnormalities, or muscle weakness, cannot be overlooked (70). Additionally, since SPN targets the peripheral nerves that innervate the spastic muscles, its efficacy may be limited in cases of widespread spasms affecting multiple muscle groups.

### 5.3 Selective dorsal Rhizotomy (SDR)

Selective dorsal rhizotomy reduces the spinal cord’s excessive excitatory output to the muscles by selectively severing the abnormal afferent nerve fibers in the dorsal roots of the spinal nerves that cause spasticity (71). It primarily targets lower limb spasticity, particularly in patients with bilateral spastic paralysis, requiring the patients to retain some degree of motor function and to be free from bodily weakness or tone disorders (72). Furthermore, this surgical procedure necessitates a laminectomy, which poses significant trauma to the patient and carries a risk of spinal instability. Postoperatively, it usually requires intensive physical rehabilitation therapy (73), which limits the range of patients who can benefit from it.

### 5.4 C7 nerve transfer

C7 nerve transfer was initially used to treat brachial plexus avulsion injuries (74). Xu et al. (75) innovatively applied this technique to children with spastic paralysis, observing a reduction in spasticity in the elbow, wrist, and second to fifth fingers, as well as an improvement in extension strength during follow-up. In recent years, C7 nerve transfer has gradually been applied in the treatment of limb spasticity following central nervous system injuries. Surgery typically involves transplanting the healthy C7 nerve root and intercostal trunk through a short conduit to the affected side, facilitating faster nerve regeneration and functional recovery (76). Multiple studies have demonstrated that patients with chronic stroke who undergo surgery exhibit significant improvements in both the Fugl-Meyer Assessment scores and the Modified Ashworth Scale scores (76, 77). However, the C7 nerve grafting procedure requires a high level of anatomical knowledge and meticulous surgical skills from the operator, making the surgery quite complex. The traditional open surgical approach requires an 8–10 cm incision in the donor site, resulting in significant trauma to the patient. To address this issue, researchers have proposed various approaches to optimize this surgical technique (78–80), aiming to reduce the length of nerve grafts, shorten the distance for nerve regeneration, and simultaneously minimize the risk of damaging the vertebral arteries. Further research suggests the use of neuroendoscopy to enhance the safety and minimally invasive nature of C7 nerve grafting. However, this approach is still in the research phase and has not yet been applied clinically (81). It is noteworthy that although many studies have demonstrated the potential of C7 nerve transfer surgery for treating limb spasticity, there is some controversy over using the healthy limb nerve as a graft recipient (82).

## 6 Neuroregulation technologies

Neuroregulation technologies, such as Transcranial Magnetic Stimulation (TMS), Transcranial Direct Current Stimulation (tDCS), Spinal Cord Stimulation (SCS), and Vagus Nerve Stimulation (VNS), have garnered increasing attention in recent years as adjuncts for rehabilitation following stroke (83, 84). These technologies aim to improve motor dysfunction, including spasticity, by modulating cortical excitability and modulating descending pathway function.

### 6.1 Transcranial magnetic stimulation (TMS)

Transcranial magnetic stimulation (TMS) is a non-invasive brain stimulation technique that has been investigated for its potential to improve various neurological dysfunctions following stroke, including motor impairment, dysphagia, cognitive deficits, aphasia, central post-stroke pain, and spasticity (83). The therapeutic rationale for spasticity management is based on rebalancing interhemispheric inhibition: low-frequency (≤1 Hz) rTMS applied to the contralesional hemisphere aims to reduce excessive transcallosal inhibition, while high-frequency (≥5 Hz) stimulation to the ipsilesional hemisphere seeks to enhance corticospinal excitability and promote neuroplasticity. The underlying mechanisms are complex and may involve neurogenesis, angiogenesis, anti-inflammatory effects, antioxidant pathways, and anti-apoptotic processes (83).

Several Clinical experiments have examined the efficacy of rTMS for post-stroke spasticity (85, 86). Lee and Ryu (87),meta-analyzed 68 RCTs, demonstrating significant upper limb spasticity reduction on the Modified Ashworth Scale (mean difference: -0.48; 95% CI -0.64 to −0.33; *p* < 0.00001); however, evidence certainty was low and lower limb data insufficient. Wang et al. (88), randomized 110 stroke patients to active rTMS (10 Hz to M1, 1 Hz to Erb’s point) or sham plus rehabilitation. Active rTMS significantly reduced MAS scores (*p* = 0.004) and improved Fugl-Meyer Upper Extremity scores (*p* < 0.05). Lahre et al. (89), demonstrated that low-frequency rTMS to contralesional M1 suppressed contralesional hemisphere activity (reduced MEP amplitude, increased latency), paralleling clinical improvements.

Current evidence supports rTMS as an adjunctive intervention for upper limb spasticity. Outcomes depend on stimulation parameters, target hemisphere, and patient characteristics (87). MEP presence may predict treatment response (89). Lower limb spasticity evidence remains insufficient (87).

### 6.2 Transcranial direct current stimulation (tDCS)

Transcranial Direct Current Stimulation modulates the resting membrane potential of cortical neurons by applying weak direct current to the scalp, thereby altering their excitability. Studies have shown that tDCS has a relatively significant effect on improving upper limb spasms. Combined with traditional rehabilitation treatment, tDCS can significantly reduce upper limb spasm in stroke patients, especially in those in the subacute and chronic phases. In addition, anodic stimulation combined with other therapies has demonstrated higher effectiveness in improving upper limb spasms, especially when the stimulation duration exceeds 20 minutes (90). However, the effect of using tDCS alone is rather limited (91). The efficacy of tDCS for lower extremity spasm remains unclear. Some studies have found that the improvement effect of single or multiple tDCS on lower extremity spasm is limited (92, 93). The therapeutic effect of tDCS is closely related to the stimulation parameters. Factors such as stimulation time, intensity, and electrode position may all affect the treatment outcome. Across tDCS studies for post-stroke spasticity, significant heterogeneity in sample sizes, study designs, and outcome measureslimits the overall reliability of treatment effect estimates (90, 91).

### 6.3 Spinal cord stimulation (SCS)

Spinal cord stimulation is further divided into epidural spinal cord stimulation (eSCS) and transcutaneous spinal cord stimulation (tSCS). eSCS involves the implantation of stimulating electrodes into the epidural space, generating electrical stimulation signals that affect the neural pathways within the spinal cord, thereby modulating muscle tone and reflex activity. In contrast, tSCS delivers electrical stimulation through surface electrodes on the skin. Research indicates that the mechanisms by which spinal cord stimulation alleviates limb spasticity encompass multiple levels, including the regulation of local spinal cord neural circuits (94), reorganization of the central nervous system (95), modulation of neurotransmitters (96), and the induction of long-term neural plasticity (97). eSCS has demonstrated significant efficacy in treating spinal cord-related spasticity, with subjective improvement rates in patients reaching up to 78%, and a notable enhancement in walking ability (98). However, eSCS also carries certain risks of complications, as prolonged retention can easily lead to concurrent infections and hardware failures (98). As a non-invasive technique, tSCS has also demonstrated remarkable effects in alleviating spasms in patients with spinal cord injuries. Research has shown that after a single session of tSCS treatment, patients experience a significant reduction in muscle tone, clonus, and spasm frequency, with effects lasting several hours (99). Furthermore, tSCS can improve patients’ daily living abilities, sleep quality, and alleviate pain symptoms (100). However, its effectiveness varies significantly among individuals (99, 100). While it demonstrates certain potential in reducing spasms, it also faces limitations due to its transient effects, necessitating long-term multi-segment stimulation (99).

### 6.4 Vagus nerve stimulation (VNS)

Vagus nerve stimulation enhances neuroplasticity by activating the nucleus tractus solitarius, which projects to noradrenergic and cholinergic nuclei, promoting synaptic reorganization and motor recovery after stroke (101–103). Preclinical studies have demonstrated that VNS reduces infarct volumes and improves neurological deficits through anti-inflammatory mechanisms, modulation of apoptotic pathways, and enhancement of angiogenesis (103). Clinical evidence supports the use of VNS in improving motor function, with significant benefits when combined with task-specific training. A 2025 meta-analysis of 18 randomized controlled trials involving 954 participants confirmed that VNS significantly improves Fugl-Meyer Assessment of Upper Extremity scores (SMD = 0.89, 95% CI 0.59 to 1.20), Wolf Motor Function Test scores, and Functional Independence Measure scores compared to control interventions (104).

Despite these promising functional improvements, evidence specifically for spasticity outcomes remains limited and of low quality. According to the Grading of Recommendations Assessment, Development and Evaluation (GRADE) framework applied in this meta-analysis, spasticity outcomes were rated as low quality, in contrast to outcomes such as swallowing function which were rated as high quality (104). A 2025 randomized controlled trial by Moustafa et al. (105) (*n* = 78) specifically examined transcutaneous auricular VNS (taVNS) combined with physical therapy in chronic stroke patients. While the intervention significantly improved neuroplasticity biomarkers (brain-derived neurotrophic factor), reduced inflammatory markers (interleukin-6), and enhanced gross manual dexterity (Box and Block Test) compared to sham stimulation, no significant difference was observed in spasticity as measured by the Modified Ashworth Scale (*p* > 0.05) (105).

However, VNS shows promise in specific rehabilitation contexts where careful patient selection is applied. Chang et al. (2021) (106), found that taVNS administered during robotic training significantly reduced spasticity of the wrist and hand at discharge as measured by the Modified Tardieu Scale (taVNS = −8.94% vs. sham = +2.97%, *p* < 0.05), with surface electromyography results demonstrating significantly increased variance for bicep peak sEMG amplitude during extension, suggesting improved antagonist control. This suggests that patients undergoing robotic-assisted rehabilitation may derive additional anti-spasticity benefits from VNS, particularly when stimulation is timed to coincide with premotor planning and task-specific training (106).

Strict adherence to patient selection criteria is essential to optimize outcomes. The presence of motor evoked potentials (MEPs) indicating preserved corticospinal connectivity may identify patients more likely to respond to VNS (101). Additionally, stroke chronicity (≥9 months post-stroke), baseline upper limb impairment severity (moderate to severe), and ability to actively participate in intensive rehabilitation are important considerations (103, 104).

In summary, while VNS does not directly reduce spasticity and evidence for anti-spasticity effects is inconsistent, it represents a promising adjunctive intervention for upper limb motor recovery in carefully selected patients undergoing robotic or task-specific rehabilitation. Future research should focus on optimizing patient selection criteria, standardizing stimulation protocols, and clarifying the conditions under which VNS may indirectly influence spasticity through enhanced motor control and reduced compensatory patterns (101, 103).

## 7 Conclusion and perspective

Early identification and intervention are essential for optimizing outcomes in post-stroke spasticity. However, current clinical scales have limitations in objectively capturing spasticity severity. Multimodal assessment systems integrating biomechanical and neurophysiological measures offer more precise evaluation but remain underutilized in clinical practice.

Botulinum toxin type A is the first-line treatment for focal spasticity, though its impact on functional improvement remains debated. Physical rehabilitation techniques including ESWT, robot-assisted training, and FES have shown generally positive results, while surgical interventions offer durable spasticity reduction for carefully selected patients.

Neuromodulation techniques including TMS, tDCS, SCS, and VNS have emerged as promising adjunctive approaches. Meta-analyses confirm that rTMS significantly reduces upper limb spasticity (mean difference: −0.48 on MAS), tDCS combined with rehabilitation improves upper limb spasticity, and SCS achieves subjective improvement rates up to 78%. VNS enhances neuroplasticity and motor recovery, though it does not directly reduce spasticity. Treatment efficacy depends critically on patient selection and stimulation parameters. Future research should prioritize large randomized controlled trials with standardized protocols, identification of biomarkers such as MEP status to guide patient selection, optimization of stimulation parameters for each neuromodulation modality, and integration of objective assessment tools to track treatment response. Addressing these priorities will be essential to translate the promise of neuromodulation into more precise, personalized, and effective spasticity management for stroke survivors.

## 8 Limitations

This review has several limitations. First, the literature search was restricted to PubMed and CNKI, excluding Embase, Scopus, and the Cochrane Library, which may introduce selection bias. Second, as a narrative review, formal quality assessment using standardized tools was not performed. Third, heterogeneity in stimulation parameters and outcome measures across studies precludes definitive conclusions regarding optimal protocols.
